# Plasma protein biomarkers of Alzheimer's disease endophenotypes in asymptomatic older twins: early cognitive decline and regional brain volumes

**DOI:** 10.1038/tp.2015.78

**Published:** 2015-06-16

**Authors:** S J Kiddle, C J Steves, M Mehta, A Simmons, X Xu, S Newhouse, M Sattlecker, N J Ashton, C Bazenet, R Killick, J Adnan, E Westman, S Nelson, H Soininen, I Kloszewska, P Mecocci, M Tsolaki, B Vellas, C Curtis, G Breen, S C R Williams, S Lovestone, T D Spector, R J B Dobson

**Affiliations:** 1 MRC Social, Genetic and Developmental Psychiatry Centre, Institute of Psychiatry, Psychology and Neuroscience, King's College London, London, UK; 2 Department of Twin Research & Genetic Epidemiology, King's College London, London, UK; 3 Centre for Neuroimaging Sciences, Institute of Psychiatry, Psychology and Neuroscience, King's College London, London, UK; 4 NIHR Biomedical Research Centre for Mental Health and Biomedical Research Unit for Dementia at South London and Maudsley NHS Foundation, London, UK; 5 Department of Old Age Psychiatry, Institute of Psychiatry, Psychology and Neuroscience, King's College London, London, UK; 6 Department of Neurobiology, Care Sciences and Society, Karolinska Instituet, Stockholm, Sweden; 7 SomaLogic, Boulder, CO, USA; 8 Institute of Clinical Medicine – Neurology, University of Eastern Finland, Kuopio, Finland; 9 NeuroCenter, Kuopio University Hospital, Kuopio, Finland; 10 Department of Old Age Psychiatry and Psychotic disorders, Medical University of Łódź, Łódź, Poland; 11 Institute of Gerontology and Geriatrics, University of Perugia, Perugia, Italy; 12 3rd Department of Neurology, Aristotle University, Thessaloniki, Greece; 13 Department of Internal Medicine and Geriatric Medicine, INSERM University of Toulouse, Toulouse, France; 14 Department of Psychiatry, Oxford University, Warneford Hospital, Oxford, UK

## Abstract

There is great interest in blood-based markers of Alzheimer's disease (AD), especially in its pre-symptomatic stages. Therefore, we aimed to identify plasma proteins whose levels associate with potential markers of pre-symptomatic AD. We also aimed to characterise confounding by genetics and the effect of genetics on blood proteins in general. Panel-based proteomics was performed using SOMAscan on plasma samples from TwinsUK subjects who are asymptomatic for AD, measuring the level of 1129 proteins. Protein levels were compared with 10-year change in CANTAB-paired associates learning (PAL; *n*=195), and regional brain volumes (*n*=34). Replication of proteins associated with regional brain volumes was performed in 254 individuals from the AddNeuroMed cohort. Across all the proteins measured, genetic factors were found to explain ~26% of the variability in blood protein levels on average. The plasma level of the mitogen-activated protein kinase (MAPK) MAPKAPK5 protein was found to positively associate with the 10-year change in CANTAB-PAL in both the individual and twin difference context. The plasma level of protein MAP2K4 was found to suggestively associate negatively (*Q*<0.1) with the volume of the left entorhinal cortex. Future studies will be needed to assess the specificity of MAPKAPK5 and MAP2K4 to eventual conversion to AD.

## Introduction

Currently, no treatments exist that delay or prevent onset of Alzheimer's disease (AD). Studies using magnetic resonance imaging (MRI) and positron emission tomography brain scans have revealed early signs of AD pathology in subjects up to ~4 and ~17 years before the onset of dementia, respectively.^1^ Pathological changes are likely to be least advanced in asymptomatic subjects, and therefore potentially the most remediable in this group. This is the motivation behind prevention trials in asymptomatic subjects with MRI or positron emission tomography evidence of early AD pathology.^2^

However, such brain scans can be relatively expensive and require specialised facilities. Therefore, researchers are looking for surrogate markers that are relatively inexpensive and non-invasive, yet informative enough for enrichment of prevention trials. Preliminary results reviewed in Lista *et al.,*^3^ Zürbig and Jahn^4^ and Chiam *et al.*^5^ suggest that blood may be a useful source of such markers. Despite this, only two discovery studies have been performed comparing blood proteins to AD-related phenotypes exclusively in asymptomatic individuals.^6, 7^

Recently, with the advent of panel-based proteomics, high-throughput studies are becoming more feasible. One such approach, SOMAscan (slow off-rate modified aptamer), allows >1000 proteins to be measured simultaneously and has already been applied in AD research.^8, 9, 10, 11^ This approach has not yet been applied to discover blood markers of AD-related phenotypes in asymptomatic individuals.

Although most blood protein biomarker studies have used cohorts of individuals, one study of *APOA1* blood DNA methylation and protein level has been performed using 24 twin pairs discordant for episodic memory performance. Two *APOA1* CpG sites were found to associate with discordant episodic memory. However, the protein level of APOA1 was not found to differ between discordant twins, possibly due to the small sample size available.^12^

An advantage of studying ‘omics' markers in twin cohorts is that it allows genetic and environmental influences on markers and traits to be studied.^13^ If the monozygotic (MZ, that is, ‘identical') twin difference in the level of a marker associates with the twin difference in trait value in twin pairs, this suggests that the relationship between marker and trait is not confounded by age, familial environment or genetics. Confounding of AD blood biomarkers by age and/or genetics is a real possibility, as these factors have been shown to affect both AD risk^14, 15^ and plasma protein levels.^16, 17, 18^ Despite this, twin differences have not yet been exploited in studies of blood protein biomarkers of AD-related phenotypes.

Genetic markers are generally more practical, robust and inexpensive to measure in comparison with blood proteins. If the association between a blood protein and an AD-related phenotype is confounded by genetics, then it is likely that a genetic test alone, rather than a protein test, would prove more cost effective as a trial enrichment biomarker. One reason to believe that genetic markers alone may not be optimal is that, it has been shown that common variants, considered additively, explain only 24–33% of the total AD variation.^19, 20^

In terms of cognitive tests, the mini mental state examination (MMSE) is known to not be very sensitive for detecting very early AD-related changes in cognition. The paired associates learning (PAL) task of the CANTAB cognition battery is thought to be more sensitive at this stage. A 4-year study by de Jager *et al.*^21^ following healthy older adults has shown that CANTAB-PAL, along with another test called the Graded Naming Test, associates with significantly higher risk of conversion to mild cognitive impairment or AD. However, the rate of CANTAB-PAL decline, which may be more informative, has not yet been evaluated in this context. It has also not yet been used as an endophenotype in a blood biomarker study. In relation to age-related cognitive decline, Steves *et al.*^22^ have shown that a larger proportion of variance in 10-year change in CANTAB-PAL total errors is explained by the non-shared environment than by additive genetics. A plasma protein biomarker measured in a single blood sample could be more practical in some settings than cognitive testing over a 10-year period in healthy older adults.

In this study, we use the TwinsUK and AddNeuroMed (ANM) SOMAscan data sets. First a heritability analysis is performed and then the association between plasma proteins and AD endophenotypes (10-year change in CANTAB-PAL and regional brain volumes) is examined in asymptomatic individuals.

## Materials and methods

### Ethics statement

Informed consent was obtained for all subjects according to the Declaration of Helsinki (1991), and protocols and procedures were approved by the relevant local ethical committees at each site.

### Discovery cohort—TwinsUK

Subjects used in this study were recruited from TwinsUK, a national register of adult twins.^23^ Full details of subject selection is given in Supplementary Methods. In brief, a total of 212 subjects (106 twin pairs) were selected with longitudinal CANTAB-PAL data (~1999 and ~2009).^22^ These subjects had also been assessed using MMSE at the ~2009 visit. Fasted EDTA plasma samples, taken a median of around 16 months before CANTAB-PAL and MMSE testing (~2009), were used for proteomic analysis, that is, around 8 years after the ~1999 assessment. Given the high level of missingness in the *APOE E4* genotype, and the small number of double*-APOE E4* carriers, we excluded double-*APOE E4* carriers from further analysis. Subjects who were homozygous for the *APOE E4* allele (10 individuals), or whose plasma sample was haemolysed (six individuals, determined by SomaLogic (Boulder, CO, USA)) or failed SOMAscan quality control (1 individual) were excluded leaving 195 subjects (93 twin pairs and 9 individuals).

### Replication cohort—AddNeuroMed

Subjects used in the replication study were individuals from the ANM cohort;^24, 25^ full details are given in Supplementary Methods and are summarised here. In total, 254 subjects with both plasma SOMAscan and MRI baseline data, excluding subjects homozygous for the *APOE E4* allele (57 individuals), were available.^9, 10^ Baseline EDTA plasma samples from fasted subjects were used; see Supplementary Table 1 for a comparison of the sample collection, processing and SOMAscan assay between TwinsUK and ANM. Plasma samples were collected within a year and a half of MMSE testing and MRI scanning. Quality control led to the exclusion of seven subjects from ANM (Supplementary Methods). The remaining subjects were diagnosed as either asymptomatic (control, *N*=91), mild cognitive impairment (*N*=81) or AD (*N*=82).

### Proteomics

SOMAscan methods and data for TwinsUK and ANM have been described previously,^18^ but are described again in Supplementary Methods, and are briefly summarised here. A single assay was used per plasma sample. All proteomics data were transformed using the natural logarithm and transformed to zero mean and unit s.d. In addition, protein values >2.5 s.d. from the mean were excluded as outliers.

### Magnetic resonance imaging

Specifics of MRI pre-processing for each cohort are described below. Owing to differences in methods, all MRI data were logged and transformed to zero mean and unit s.d. to increase comparability.

#### TwinsUK

TwinsUK MRI scans were performed ~2 years after plasma sampling (median 805 days, interquartile range (IQR) 151 days). Volumes of the hippocampi and the combined Brodman's areas 28 and 34 (equivalent to the entorhinal cortex) in the TwinsUK cohort were obtained from 38 subjects using Diffeomorphic Anatomical Registration through Exponentiated Lie Algebra,^26^ Statistical Parametric Mapping^27^ and MarsBar,^28^ as fully described in Supplementary Methods.

#### AddNeuroMed

Volumes of the hippocampi and entorhinal cortices in the ANM cohort were obtained using FreeSurfer 5.1.0 (freely available from http://surfer.nmr.mgh.harvard.edu/) from 276 ANM subjects who had undergone structural MRI. These regions were selected as they are known to be related to early AD pathology, and were normalised by intracranial volume.^29^ Detailed information about data acquisition, pre-processing and quality control assessment have been described for this cohort in detail elsewhere.^29, 30, 31, 32, 33^

### APOE genotyping

Full details are given in Supplementary Methods, but in brief single-nucleotide polymorphisms rs429358 and rs7412 were determined from DNA samples using a TaqMan assay.

### Statistical analysis

A detailed account of the statistical methodology is provided in Supplementary Methods, and is summarised here. All statistical analyses were performed in R 3.1.0, except for the transformation and 10-year change calculations for cognitive scores that was performed in STATA 11.^22^ All double-*APOE E4* carriers were excluded from analyses. Regressions in the discovery cohort were performed using generalised estimation equations to account for twin dependencies.^34, 35^ Subject age, gender and recruitment centre were used as co-variates in all relevant regressions. The Benjamini–Hochberg (that is, false discovery rate) multiple testing correction was used to generate *Q*-values with thresholds of *Q*<0.05 used to indicate association and *Q*<0.1 to indicate suggestive association.

Analysis of association between a protein level and 10-year change in CANTAB-PAL in the twin difference context was performed by calculating twin differences in both, and performing a linear regression between the two differences, covarying for twin-pair age. Twin modelling was performed using structural equation modelling to estimate the proportion of variance explained by additive genetics (A), shared environment (C) and non-shared environment (E).^36^

## Results

### Characteristics of the TwinsUK-SOMAscan subcohort

Characteristics of the TwinsUK subjects with SOMAscan data are summarised in Table 1. The MZ and dizygotic twins are matched well, with the exception of *APOE* missingness. Despite not being clinically diagnosed as cognitively impaired, there is variability in the cognitive ability of these subjects (MMSE range 23–30). The subject with MMSE score of 23 was included, as they showed no functional deficit and did not affect the results of the following analyses (data not shown); in all other subjects the MMSE score was >25.

### Heritability of plasma protein levels in TwinsUK

Twin modelling was used to estimate the heritability of plasma proteins measured by SOMAscan (Supplementary Table 2). The median proportion of variance of plasma protein levels explained by additive genetics (A) was found to be 26% (IQR 3–46%). The proportion explained by total familial factors (additive genetics (A) or twin-shared environment (C)) was 34% (IQR 3–74%). Finally, a median of 54% (IQR 40–75%) of the variance in plasma protein levels was found to be explained by non-shared environmental factors (E), including technical variability. The results were comparable when a Van der Waerden transformation was used to overcome deviations from the normal distribution (Supplementary Table 2).

### Plasma protein markers of cognitive scores in asymptomatic individuals from TwinsUK

Levels of plasma proteins in TwinsUK subjects were first compared with MMSE scores, both as a continuous and dichotomised variable. No protein was found to be associated in either analysis (Supplementary Tables 4–5).

**Figure 1:**
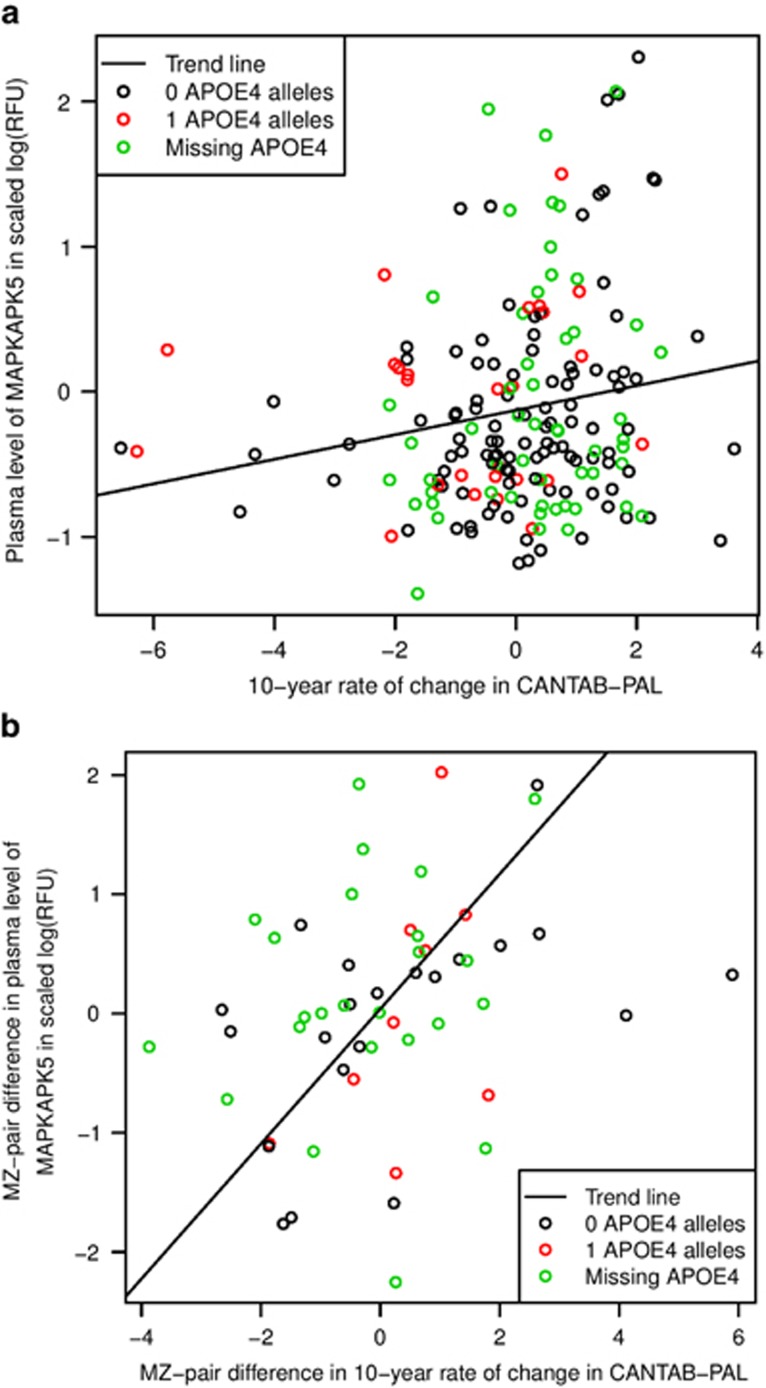
The relationship between plasma MAPKAPK5 levels and 10-year change in CANTAB-PAL in TwinsUK. Scatterplot comparing (**a**) plasma MAPKAPK5 levels and 10-year change in CANTAB-PAL in TwinsUK, or (**b**) MZ twin differences in both. Points are coloured by *APOE E4* data. (**a**) GEEs covarying for age and taking into account twin relatedness show an association between plasma MAPKAPK5 and the 10-year change in CANTAB-PAL (*β*=0.48, *Q*=0.0059). (**b**) Linear regression covarying for age shows an association between plasma MAPKAPK5 and the 10-year change in CANTAB-PAL in the MZ twin difference context (*β*=0.55, *P*=0.030). CANTAB-PAL, CANTAB-paired associates learning; GEE, generalised estimation equation; MZ, monozygotic; RFU, relative fluorescence unit.

No protein passed multiple testing corrections for association with CANTAB-PAL total errors in 1999 or 2009; however, MAPKAPK5 was found to be associated with the 10-year change in CANTAB-PAL total errors (Figure 1a; *β*=0.48, *Q*=0.0059; Supplementary Tables 5–7).

In total, 54 MZ twin pairs had complete data on plasma MAPKAPK5 levels and 10-year change in CANTAB-PAL total errors. Within these subjects, MAPKAPK5 was found to associate with 10-year change in CANTAB-PAL total errors in the context of MZ-pair differences (Figure 1b; *β*=0.55, *P*=0.030). In a twin model, non-shared environmental factors (E) were found to explain 90% (95% confidence interval (CI): 64–100%) of the variance in the plasma level of MAPKAPK5 when a Van der Waerden transformation was applied (A=10% (CI: 0–36%), C=0% (CI: 0–23%) and E=90% (CI: 64–100%); Supplementary Table 1).

### Plasma protein markers of MRI measures in asymptomatic individuals from TwinsUK

Plasma protein levels were then compared with selected regional brain volumes derived from structural MRI scans, taking into account age and twin relatedness (Supplementary Tables 8–11). These selected regions of interest—the left and right hippocampus and entorhinal cortices (LE and RE, respectively)—are known to show early pathology and greater atrophy in AD.^1, 37^

**Figure 2:**
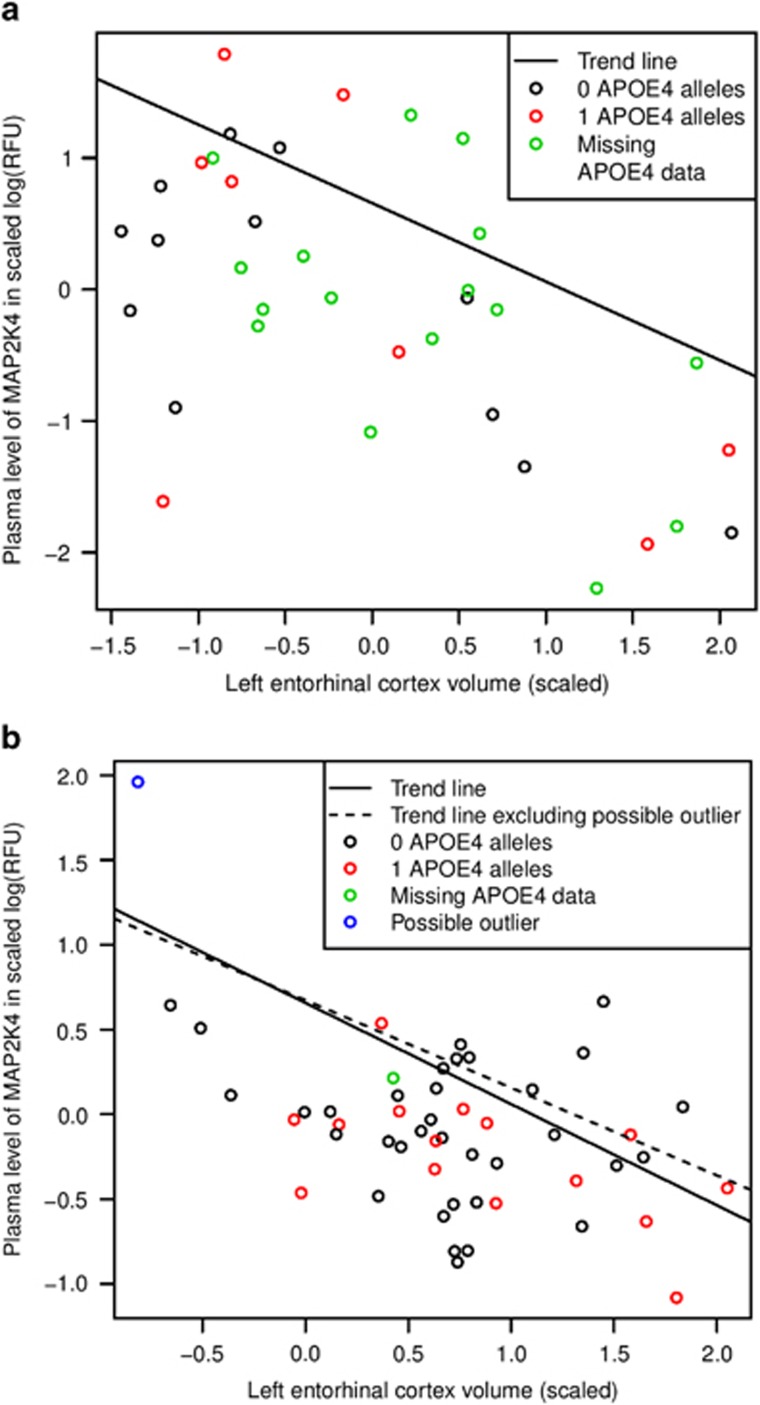
The association between plasma MAP2K4 levels and (**a**) left parahippocampal volume in TwinsUK or (**b**) left entorhinal cortex volume in ANM. Scatterplot comparing plasma MAP2K4 levels with the volume of the left entorhinal cortex are given for the two cohorts. Points are coloured by *APOE E4* data and whether they appear to be outliers. (**a**) GEEs covarying for age and taking into account twin relatedness show a suggestive association (*Q*<0.1) between plasma MAP2K4 and the volume of the left entorhinal cortex in TwinsUK. (**b**) Linear regression covarying for age and centre shows a suggestive association (*Q*<0.1) between plasma MAP2K4 and the volume of the left entorhinal cortex in ANM, when a possible outlier (in blue) is included (*β*=−0.64, *P*=0.0025 and *Q*=0.088), or nominal association (*P*<0.05) when it is excluded (*β*=−0.54 and *P*=0.014). ANM, AddNeuroMed; GEE, generalised estimation equation.

Full results are given in Supplementary Tables 8–11. The plasma level of one protein—FAM107B—was found to associate with the volume of both the LE (*β*=1.05, *Q*=0.034) and RE (*β*=0.77, *Q*=0.015) cortices. Two proteins associated with the volume of one region (*Q*<0.05) were also associated with the volume of three out of four regions at a suggestive threshold of *Q*<0.1, NSF1C (positively with left hippocampus, LE and RE) and MAP2K4 (negatively with right hippocampus, LE and RE; Supplementary Tables 8–11). The association between MAP2K4 and LE cortex volume in TwinsUK is shown in Figure 2a.

Of the three proteins most consistently associated with multiple brain regions—FAM107B, NSF1C and MAP2K4—only MAP2K4 was also found to be nominally associated with the 10-year change in CANTAB-PAL total errors, (rank=16th, *β*=−0.24, *P*-value=0.026, *Q*=~1.0; Supplementary Table 7). Similarly, MAPKAPK5 was nominally associated with the volume of the right hippocampus (rank=93rd, *β*=−0.28, *P*-value=0.039, *Q*=0.48; Supplementary Table 6).

### Replication of discovery results in ANM

We wished to replicate the association of plasma levels of the proteins FAM107B, NSF1C and MP2K4 with MRI regional volumes in the ANM SOMAscan data. However, as TwinsUK consists of asymptomatic females, whereas ANM also includes males and cognitively impaired individuals, we wished to see whether this had any impact on replication. We therefore performed the replication analysis in ANM in all subjects, in just the asymptomatic (control) subjects and in just the asymptomatic females separately. The characteristics of the relevant ANM subcohorts are given in Table 2.

The plasma level of the protein MAP2K4 in asymptomatic female ANM subjects was found to suggestively associate with the volume of LE (*β*=−0.64, *P*=0.0025, *Q*=0.088), in a direction consistent with the discovery cohort (Figure 1). Although this trend is visible in Figure 2b, there is a possible outlier remaining in the data set (with the lowest volume and highest protein level). Removing this possible outlier reduces the significance but does not remove the association of MAP2K4 with the volume of LE in asymptomatic females (*β*=−0.54, *P*=0.014).

No other combination of proteins, subjects or regions showed an association (Supplementary Table 12). However, the directions of association were consistent for all the proteins between asymptomatic females from ANM and TwinsUK, and the models including these proteins appeared to explain a higher proportion of the variance (*R*^2^) in asymptomatic females versus the other subject groups.

Twin models suggest that plasma levels of MAP2K4 may be more affected by additive genetics than by environmental influences (A=72% (CI: 15–83%), C=0% (CI: 0–55%) and E=28% (CI: 17–39%); Supplementary Table 1).

## Discussion

In this study we have applied the following methodologies to the study of blood protein biomarkers of AD endophenotypes for the first time: (1) the use of panel-based proteomics in an asymptomatic cohort, (2) the use of CANTAB-PAL as an endophenotype and (3) the use of twin samples. We also believe this is the third discovery study of blood protein markers of AD endophenotypes in asymptomatic individuals, and the largest twin high-throughput proteomics study performed to date.

We are only aware of one other study estimating the heritability of blood protein levels in a high-throughput manner.^17^ The estimates from our study suggest a heritability that is at least twice as high on average, further demonstrating the potential for genetic confounding of blood protein biomarkers.

The suggestive/nominal replication of MAP2K4 in ANM, again in asymptomatic individuals, is consistent with the hypothesis that it shows the greatest variability in the asymptomatic phase of AD, which is what is required of an early biomarker. This may be why it has not been reported in previous AD blood biomarker studies, which have mostly focused on later disease stages. As well as being negatively associated with entorhinal cortex volume, MAP2K4 has been linked to phosphorylation of tau^38^ and of amyloid precursor protein through c-Jun N-terminal kinase.^39^ In both cases, these phosphorylation events are believed to lead to increased AD pathology.

MAPKAPK5 has mostly been studied in the context of cancer^40^ and rheumatoid arthritis,^41^ and, to the best of our knowledge, has not previously been implicated in AD. However, it is interesting that we find two different mitogen-activated protein kinases (MAPKs) whose levels in plasma associate with AD endophenotypes.

The use of longitudinal change in CANTAB-PAL, a cognitive test that is sensitive to early cognitive decline, was critical in the discovery of MAPKAPK5 as a potential biomarker relevant to AD. This demonstrates the importance of studying decline in cognitive abilities using sensitive tests in asymptomatic older adults.

By showing an association between MAPKAPK5 and 10-year change in CANTAB-PAL total errors in the MZ twin difference context, we have demonstrated that the association of MAPKAPK5 and change in cognitive ability in older adults is independent of genetic and twin-shared environmental factors. This is consistent with twin modelling, which suggests that plasma MAPKAPK5 levels are mostly affected by non-shared environmental factors. This has also been demonstrated for 10-year change in CANTAB-PAL total errors in a previous study.^22^ Genetic risk, and many aspects of twin-shared environment (such as maternal and family factors) have been implicated in cognitive ageing and dementia, but are currently not modifiable. Given our findings, we hypothesise that MAPKAPK5 may be a biomarker of modifiable cognitive ageing. Selection of individuals for intervention studies whose risk is modifiable may lead to improved outcomes.

The failed replication of findings for proteins FAM107B and NSFL1C may indicate that the original findings are false positives, or they may indicate failed replication due to the technical differences or small sample sizes present in this study. However, in asymptomatic females of the replication cohort, the directions of association of all of the proteins were consistent with discovery findings. In addition, the proportion of variance explained by the models was largest in the asymptomatic females. This is consistent with a failure of replication due to small sample size and therefore insufficient statistical power. This suggests that the sample size of this replication cohort may have been a limitation.

There is great demand for inexpensive and relatively non-invasive markers that could be used to identify asymptomatic subjects at risk of AD to recruit into prevention trials. In this study, we started by demonstrating heritability of plasma protein levels, which raise the potential of genetic confounding of plasma protein biomarkers of disease. We then looked for plasma protein biomarkers of AD endophenotypes in asymptomatic twins. The plasma protein level of two MAPKs—MAP2K4 and MAPKAPK5—were identified as possible biomarkers of early AD. MAPKAPK5 was shown to be associated with decline in cognitive ability in the context of MZ twin differences. This suggests that it conveys information on cognitive ability that is complementary to genetic markers, and may be a biomarker of modifiable cognitive ageing. Future studies will need to assess the specificity of MAPKAPK5 and MAP2K4 to eventual AD, and their potential utility as enrichment biomarkers for clinical trials.

## Figures and Tables

**Table 1:** Characteristics of the discovery cohort (TwinsUK) with SOMAscan data.

	*Monozygotic subjects*	*Dizygotic subjects*	*Subjects without twin-pair data*
Number of subjects	110 (55 pairs)	76 (38 pairs)	9
Number of MRI scan subjects	34	0	2
Age (median (IQR))	65 (62–71)	63 (59–68)	67 (63–74)
% Female	100	100	100
*APOE E4* (# alleles 0/1 {NA})	44/18 {48}	61/9 {6}	8/0 {1}
MMSE (median (IQR) {# NA})	29 (29–30) {7}	29 (29–30) {2}	28 (27–30)

Abbreviations: IQR, interquartile range; MMSE, mini mental state examination; MRI, magnetic resonance imaging; NA, individuals with missing data for this measure.

Double-*APOE E4* carriers were excluded from this study.

**Table 2:** Characteristics of the replication cohort (ANM) with complete SOMAscan and MRI data.

	*All*	*Controls only*	*Female controls only*
Number of subjects	254	91	51
% Female	100%	100%	100%
Median age (years)	74	72	72
IQR age	70–78	68–76	67–76
*APOE E4* (# alleles 0/1 {NA})	158/90 {6}	66/24 {1}	35/15 {1}
Diagnostic groups (control/MCI/AD)	91/81/82	91/–/–	51/–/–
Median MMSE by diagnostic group (control/MCI/AD)	29/28/22	29/–/–	30/–/–
IQR MMSE (control/MCI/AD)	29–30 / 26–29 / 17 - 25	29–30 /–/–	29–30 /–/–

Abbreviations: AD, Alzheimer's disease; ANM, AddNeuroMed; IQR, interquartile range; MCI, mild cognitive impairment; MMSE, mini mental state examination; MRI, magnetic resonance imaging; NA, individuals with missing data for this measure.

Double-*APOE E4* carriers were excluded from this study.
