# Supplementary material for: Plasma protein biomarkers of Alzheimer's disease endophenotypes in asymptomatic older twins: early cognitive decline and regional brain volumes
Source: Transl Psychiatry. 2015 Jun 16;5(6):e584–. doi: 10.1038/tp.2015.78 (PMC4490288; doi:10.1038/tp.2015.78)
Supplement: Supplementary Information [file tp201578x1.doc]

**Supplementary Methods**

**1 - Discovery cohort – TwinsUK**

Subjects used in this study were recruited from TwinsUK, a national register of adult twins [23]. Specifically, a subset of the subjects from a previous study of cognitive ability were used [22]. A total of 212 subjects (106 twin pairs) were selected: all of the monozygotic twin-pairs and a random selection of the dizygotic twin-pairs (chosen by R function ‘sample’) from the cohort of 324 TwinsUK subjects with longitudinal CANTAB-PAL data (~1999 and ~2009) [22]. These subjects had also been assessed using MMSE at the ~2009 visit. Fasted EDTA plasma samples, taken a median of a year and four months (median 488 days, interquartile range (IQR) of 568 days) before CANTAB-PAL and MMSE testing (~2009), were used for proteomic analysis. See Supplementary Table 1 for technical details of blood sampling and processing. Subjects who were homozygous for the APOE E4 allele (10 individuals), or whose plasma sample was haemolysed (6 individuals) or failed SOMAscan quality control (QC; 1 individual) were excluded leaving 195 subjects (93 twin-pairs and 9 individuals).

**2 - Replication cohort – AddNeuroMed**

Subjects used in the replication study were individuals from the ANM cohort [24,25]. 274 subjects with both plasma SOMAscan and MRI baseline data were available [8,9]. Subject homozygous for the APOE E4 allele were excluded, leaving a total of 254 individuals. Baseline EDTA plasma samples from fasted subjects were used, see Supplemental Table 1 for a comparison of the sample collection, processing and SOMAscan assay between TwinsUK and ANM. Plasma samples were collected within a year and a half of MMSE testing and MRI scanning. All subjects were assessed with a standardized assessment protocol including an informant interview for diagnosis and cognitive assessment such as the MMSE, which has been described previously [24,25]. Subjects were diagnosed as either asymptomatic (control, N = 91), Mild Cognitive Impairment (MCI, N = 81) or AD (N = 82) based on National Institute of Neurological and Communicative Disorders and Stroke and the Alzheimer’s Disease and Related Disorders Association (NINCDS- ADRDA) criteria.

**3 - Proteomics**

SOMAscan methods and data for TwinsUK and ANM have been described previously [18], but are described again briefly here. Proteins were measured using a SOMAmer-based capture array called ‘SOMAscan’ (SomaLogic, Inc, Boulder, Colorado). For more details on the assay see Gold et al [11] and the SomaLogic Inc website (http://www.somalogic.com/Products-Services/SOMAscan/FAQs.aspx).

A single assay was used per plasma sample, i.e. no technical replicates were performed. TwinsUK proteomic data was collected using SOMAscan version 3 on 65 μl of plasma, measuring 1,129 proteins, whereas ANM+ARUK+DCR proteomic data was collected using SOMAscan version 2 on 8 μl of plasma, measuring 1,001 proteins. All except 21 of the proteins measured by SOMAscan v2 are also measured by SOMAscan v3. TwinsUK proteomics data are publicly available upon request on the department website (http://www.twinsuk.ac.uk/data-access/accessmanagement/). We aim to make the ANM+ARUK+DCR data available to the public as soon as possible. In the short term the authors can be contacted and collaborations established under an agreement shared with SomaLogic.

SomaLogic’s standard QC and normalization processes were applied [18]. Data from one sample from the TwinsUK cohort failed SomaLogic’s QC procedures and so was excluded. Six additional TwinsUK samples (two from subjects who had undergone MRI scans) appeared to be haemolysed, and so were excluded. No Principal Component Analysis (PCA) outliers were identified for the TwinsUK SOMAscan data, whereas 7 PCA outliers from the ANM+ARUK+DCR SOMAscan data were identified and excluded. All proteomics data was transformed using the natural logarithm and transformed to zero mean and unit standard deviation SD. Additionally, protein values >2.5 SD from the mean were excluded as outliers. This cutoff was chosen as a lower cutoff produced low p-values for some proteins which appeared to be driven by outliers.

**4 – TwinsUK Magnetic Resonance Imaging**

TwinsUK MRI scans were performed approximately two years after plasma sampling and MMSE testing (median 805 days, IQR 151 days). Volumes of the hippocampi and the combined Brodman’s areas 28 and 34 (equivalent to the entorhinal cortex) in the TwinsUK cohort were obtained from 38 subjects using the Diffeomorphic Anatomical Registration through Exponentiated Lie Algebra (DARTEL) technique [26]. This method produces a template based on this population of older female brains leading to improved registration. Structural processing was performed using Statistical Parametric Mapping (SPM8) software (http://www.fil.ion.ucl.ac.uk/spm/software/spm8). The structural magnetic resonance images were converted to axial slices and origins of all images reset to the anterior commissure, prior to segmentation using the standard unified segmentation model in SPM8 [27]. Then, Grey Matter (GM) population templates were generated from the entire image dataset using the DARTEL technique [26]. After an initial affine registration of the GM DARTEL templates to the tissue probability maps in Montreal Neurological Institute (MNI) space (http://www.mni.mcgill.ca/), non-linear warping of GM images was performed to the DARTEL GM template in MNI space with a 1.5mm cubic resolution. The GM volume (GMV) at each voxel was obtained through modulation. Finally, the GMV images were smoothed with Gaussian kernel with a Full-Width at Half-Maximum of 8mm. After spatial pre-processing, GMV for the regions of interest were extracted from the smoothed, modulated and normalized images using MarsBar software [28].

**5 - APOE genotyping**

DNA was extracted from whole blood samples from TwinsUK samples using Nucleon BACC3 Genomic DNA Extraction Kits (GE Healthcare, Buckinghamshire). DNA was extracted from blood leukocytes from ANM samples by a standard phenol-chloroform extraction. The SNPs rs429358 and rs7412 were determined by allelic discrimination assays based on fluorogenic 59 nuclease activity, and the alleles inferred. APOE data for subjects with ambiguous genotypes were treated as missing. TaqMan SNP genotyping assays were performed on an ABI Prism 7900HT and analyzed using SDS software, according to the manufacturer’s instructions (Applied Biosystems, Warrington, UK).

**6 - Statistical analysis**

All statistical analyses were performed in R 3.1.0, except for the transformation and 10-year change calculations for cognitive scores. All double APOE e4 carriers were excluded from analyses, as too few were present in the discovery cohort to make inclusion as a co-variate viable. Regressions in the discovery cohort were performed using Generalised Estimation Equations (GEE), allowing twin dependencies to be accounted for as clusters with exchangeable correlation structures [34,35]. GEE analysis was performed using the ‘geepack’ package. For MMSE, due to skewness, GEE was also performed against dichotomised MMSE scores (29-30 vs 23-28). Linear regressions were performed using the ‘lm’ command. Subject age, gender, and recruitment centre were used as co-variates in all regressions, except in TwinsUK were a single centre was used, and the female only subcohort analyses where gender was not relevant. Skewness was calculated using the ‘e1071’ package. The Benjamini-Hochberg (i.e. False Discovery Rate) multiple testing correction was used to generate Q-values using ‘p.adjust’, with thresholds of Q < 0.05 used to indicate association and Q < 0.1 to indicate suggestive association. All SOMAscan analyses were performed using all proteins, except replication and twin difference analyses, which focused on candidates to increase statistical power.

Analysis of association between a protein level and 10-year change in CANTAB-PAL in the MZ-twin difference context was performed by calculating twin differences in both, and performing a linear regression between the two differences, covarying for twin-pair age.

Twin modeling was performed using Structural Equation Modelling in OpenMX [36] in R to estimate the proportion of variance explained by additive genetics (A), shared environment (C) and non-shared environment (E). This was performed on the SOMAscan data, both untransformed, and transformed using the Van der Waerden transformation (script provided by Maciej Trzaskowski).

STATA 11 was used for square root transformation of CANTAB-PAL total errors, and to calculate the 10-year change in CANTAB-PAL total errors as described in Steves et al [22]. It was also used to calculate 10-year change as a residual of a linear model, co-varying for baseline score [22].
