# Supplementary material for: Plasma protein biomarkers of Alzheimer's disease endophenotypes in asymptomatic older twins: early cognitive decline and regional brain volumes
Source: Transl Psychiatry. 2015 Jun 16;5(6):e584–. doi: 10.1038/tp.2015.78 (PMC4490288; doi:10.1038/tp.2015.78)
Supplement: Supplementary Table Legends [file tp201578x3.doc]

Supplementary Table 1 – Differences in plasma sampling, processing and SOMAscan assays between TwinsUK and ANM. Important sampling and processing variables are listed in line with the recommendations of the Blood-Based Biomarker Interest Group (BBBIG) and the STandards for Alzheimer's Research in Blood biomarkers group (STAR-B) O’Bryant et al (Ahead of print; Alzheimer’s & Dementia). Consistent steps include the use of fasted subjects, 21G or 23G needles depending on vein size, purple topped EDTA tubes, and approximately two hours from blood draw to samples freezing. Additionally the SOMAscan assay was performed on ANM, DCR and ARUK samples together, but separately for TwinsUK. RT = Room Temperature.

Supplementary Table 2 – Twin modeling results for plasma protein levels in TwinsUK. Twin models in OpenMX were applied to estimate the proportion of variance explained by (A) additive genetics, (C) shared environment and (E) non-shared environment. A cross reference between analytes and a review of blood based protein markers of AD-related phenotypes is given [5]. Twin modelling performed on pre-processed SOMAscan data, and also on SOMAscan data after a Van der Waerden normalisation which normalises data. Shapiro-Wilks tests performed on SOMAscan data to indicate deviations from normal distribution.

Supplementary Table 3 – Results from regressions of plasma proteins against MMSE score in TwinsUK. Regressions performed using GEE to account for twin dependancies, with age as a covariate.

Supplementary Table 4 – Results from regressions of plasma proteins against dichotomized MMSE score (29-30 vs 23-28) in TwinsUK. Regressions performed using GEE to account for twin dependancies, with age as a covariate.

Supplementary Table 5 – Results from regressions of plasma proteins against square root of CANTAB-PAL total errors from ~1999 in TwinsUK. Regressions performed using GEE to account for twin dependancies, with age as a covariate.

Supplementary Table 6 – Results from regressions of plasma proteins against square root of CANTAB-PAL total errors from ~2009 in TwinsUK. Regressions performed using GEE to account for twin dependancies, with age as a covariate.

Supplementary Table 7 – Results from regressions of plasma proteins against 10-year change in CANTAB-PAL total errors in TwinsUK. Regressions performed using GEE to account for twin dependancies, with age as a covariate. 10-year change in CANTAB-PAL adjusted to take into account baseline score in a linear regression.

Supplementary Table 8 – Results from regressions of plasma proteins against the volume of the left hippocampus in TwinsUK. Regressions performed using GEE to account for twin dependancies, with age as a covariate.

Supplementary Table 9 – Results from regressions of plasma proteins against the volume of the right hippocampus in TwinsUK. Regressions performed using GEE to account for twin dependancies, with age as a covariate.

Supplementary Table 10 – Results from regressions of plasma proteins against the volume of the left entorhinal cortex in TwinsUK. Regressions performed using GEE to account for twin dependancies, with age as a covariate.

Supplementary Table 11 – Results from regressions of plasma proteins against the volume of the right entorhinal cortex in TwinsUK. Regressions performed using GEE to account for twin dependancies, with age as a covariate.

Supplementary Table 12 – Results of replication study of plasma protein markers of regional brain volumes in AddNeuroMed. Regressions performed using linear regression, taking into account age, gender and recruitment centre as appropriate. The plasma levels of FAM107B, NSFL1C and MAP2K4 are compared to regional brain volumes in ANM. Three subsets of the ANM cohort with relevant data were used: All = all subjects, Ctl = only symptomatic individuals (i.e. controls), and Both = only asymptomatic females. LH = Left Hippocampus, RH = Right Hippocampus, LE = Left Entorhinal cortex and RE = Right Entorhinal cortex.
